# Constructed-Response Assessments Show Greater Differentiation by Learning Approach Than Selected-Response Assessments in Formative Undergraduate Medical Physiology

**DOI:** 10.7759/cureus.108624

**Published:** 2026-05-11

**Authors:** Mohammed Ismail-Khan, Ratna Kumari, Sanghamitra Panda, Shaik K Abdullah, Mona M Abdalla

**Affiliations:** 1 School of Medicine, University of Sunderland, Sunderland, GBR; 2 Department of Gynaecological Oncology, Northern Gynaecological Oncology Centre, Queen Elizabeth Hospital, Gateshead, GBR; 3 Department of Physiology, Shadan Institute of Medical Sciences, Hyderabad, IND; 4 Department of Physiology, Rangaraya Medical College, Kakinada, IND; 5 Department of Physiology, Malla Reddy Medical College for Women, Hyderabad, IND; 6 Department of Physiology, International Medical University, Kuala Lumpur, MYS

**Keywords:** constructed-response assessment, deep learning approach, descriptive assessment, multiple-choice questions, selected-response assessment, surface learning approach, undergraduate and graduate medical education

## Abstract

Introduction

Assessment format may influence the extent to which student learning approaches are reflected in performance, yet whether constructed-response descriptive assessments (DAs) and selected-response multiple-choice question assessments (MCQAs) differ in their sensitivity to variation in learning approach within formative physiology education has not been directly examined. This study tested whether baseline deep and surface learning approaches were differentially associated with performance in DAs and MCQAs within a longitudinal formative undergraduate medical physiology programme.

Methods

This three-month longitudinal observational study was conducted at a single medical college in South India. Among 150 invited first-year medical students, 109 completed the baseline Revised Two-Factor Study Process Questionnaire (R-SPQ-2F) and contributed to the study. Eight physiology topics, selected through a modified Delphi process, were taught sequentially and assessed on a rolling basis. For each topic, students completed both a DA and an MCQA in the same sitting, with items matched on construct and revised Bloom's taxonomy level. A linear mixed-effects model was used to test whether the association between learning approach and marks differed by assessment format, with a student-level random intercept to account for repeated observations. A post hoc inter-rater reliability audit was conducted on a randomly selected subset of DA scripts. Sensitivity analyses included a random-slopes model and a Deep-minus-Surface composite parameterisation.

Results

The association between learning approach and marks differed by assessment format, with significant format-by-deep-learning and format-by-surface-learning interactions (both p < 0.001). Within DAs, higher deep-learning scores were associated with higher marks (β = 0.032, p = 0.022), whereas higher surface-learning scores were associated with lower marks (β = -0.038, p = 0.003). Within MCQAs, neither learning-approach dimension was significantly associated with marks. The negative association between surface learning and DA performance remained robust across model specifications, whereas the positive association between deep learning and DA performance was attenuated in the random-slopes model (p = 0.072). The Deep-minus-Surface sensitivity analysis supported the same overall format-dependent pattern. A post hoc inter-rater reliability audit on 25% of DA scripts yielded strong agreement with an intraclass correlation coefficient (ICC) of 0.87.

Conclusions

Assessment format moderated the association between learning approach and assessment performance in this cohort: DAs showed clearer differentiation by learning approach than MCQAs, with poorer DA performance at higher surface-learning scores. These findings do not show that MCQAs reward surface learning but suggest that DAs may provide more discriminating information about variation in learning approach than MCQAs within a programmatic assessment framework.

## Introduction

Medical physiology is widely recognised for its central role in developing competent physicians [1,2]. It is inherently a concept-rich discipline, requiring learners to integrate and apply principles to clinical problem-solving [1,3,4]. Rote memorisation of isolated facts is insufficient for this purpose [1,3]. To prepare for clinical practice, students must therefore develop a deep, conceptual understanding of physiological principles [2,3].

The Students’ Approaches to Learning framework describes how learners engage with academic tasks and can be used to characterise deep versus surface approaches to study [5-7]. A surface learning approach is characterised by a focus on memorisation and reproduction of learning tasks to meet minimum course requirements, often hindering the ability to apply knowledge to clinical problem-solving [5,6]. In contrast, a deep learning approach is characterised by attempts to comprehend, conceptualise, and critically analyse the learning material, thereby enabling transferability and application to novel clinical situations [5,6]. For a conceptually demanding discipline such as physiology, a deep learning approach is especially desirable because it better supports conceptual mastery and transfer.

The adoption of a surface or deep learning approach is not a fixed learner characteristic but is influenced by factors within the educational environment, with assessment exerting a particularly powerful effect [5-8]. Assessment has long been recognised as a principal driver of learning behaviour [9], with Biggs and Tang emphasising that the design of an assessment largely determines whether students adopt surface or deep learning approaches [5]. Formative assessments (assessments for learning) are especially important in this regard. When appropriately designed, they can provide actionable feedback and support earlier self-regulated modification of learning approach, unlike summative assessments, which primarily serve evaluative purposes at the end of a learning period [10,11].

From the perspective of the educational consequences of assessment design, it is important to select assessment formats (AF) that can reveal meaningful differences in the quality of students’ learning [12]. Assessments that reward rote recall alone risk driving students towards adopting a surface learning approach [5]. While various AFs have context-specific utility [9,13], in formative physiology education, AFs may differ in the cognitive processes they elicit, even when targeting the same content and knowledge level, and may therefore differ in their sensitivity to meaningful variation in learning approach. A format that requires learners to construct and articulate their reasoning may be more likely to reward conceptual understanding than one that requires selection from predefined options. This could be because the act of generating a response draws more heavily on the integrative and analytical processes associated with deep learning [8]. If so, a formative AF requiring students to generate and articulate reasoning should be more sensitive to variation in learning approach than one based primarily on option recognition. The feedback it produces should also better discriminate between more deep-oriented and more surface-oriented engagement.

Written assessment of knowledge remains central in undergraduate medical education and can be classified as constructed-response or selected-response AFs. The constructed-response formats, here termed descriptive assessments (DAs), include instruments such as short-answer questions and essays, in which students generate their own responses. The selected-response formats, represented in this study by multiple-choice question assessments (MCQAs), require students to choose from predefined options. Both formats are widely used in health professions education, and while constructed-response formats can capture reasoning and synthesis, selected-response formats are often preferred for standardisation, scalability, and lower marking burden [13].

In our setting, undergraduate medical physiology students undertake DAs and MCQAs as part of longitudinal formative assessments within a programmatic framework. Existing evidence suggests that MCQ-based assessment contexts are more often associated with a surface learning approach (memorisation), whereas constructed-response contexts are more often associated with a deep learning approach (analysis and synthesis) [5,14,15].

This study examined whether students' baseline deep and surface learning approaches were differentially associated with performance in DAs and MCQAs within a longitudinal formative physiology programme. The objective was to test whether the assessment format modified the relationship between learning approach and marks.

## Materials and methods

Study design, setting, and participants

This observational study is reported with reference to the Strengthening the Reporting of Observational Studies in Epidemiology (STROBE) guidelines. This was a three-month longitudinal observational study conducted at Shadan Institute of Medical Sciences, a National Medical Commission-approved medical college in South India. All 150 first-year undergraduate medical students enrolled in the 2024-2025 academic year were invited to participate. Of these, 116 provided written informed consent. Seven students were excluded before analysis because they did not complete the baseline Revised Two-Factor Study Process Questionnaire (R-SPQ-2F) [7], leaving a complete-case analytic sample of 109 students. Because this was a whole-cohort educational study, all eligible students were invited rather than sampled from a prespecified recruitment target.

Ethical approval

The study was approved by the Institutional Ethics Committee of Shadan Institute of Medical Sciences (reference no. 02/SIMS/IEC/2024 of EC/NEW/ISNT/2023/4198). Written informed consent was obtained from all participants before data collection. Participation was voluntary and did not affect students’ grades. Responses from the study-process questionnaire (R-SPQ-2F) were not accessible to teaching staff involved in marking.

Operationalisation of the learning approach

At baseline, students completed a paper-based version of the R-SPQ-2F, a 20-item self-report questionnaire comprising 10 deep-approach items and 10 surface-approach items, each scored on a five-point Likert scale [7]. The questionnaire was administered in a supervised classroom setting, and students completed it independently without discussion with peers. Students were informed that there were no right or wrong answers and were asked to respond according to their usual approach to studying. The R-SPQ-2F is published in the original validation study and is available for research use, provided that the original source is acknowledged [7]. It was used on this basis in the present study. Separate total scores were calculated for deep and surface learning approaches and used as continuous covariates in the analysis. Internal consistency of the two R-SPQ-2F subscales was evaluated in the analytic cohort using Cronbach’s alpha. Because alpha has recognised limitations, these estimates were interpreted as descriptive indices of internal consistency rather than as evidence of unidimensionality. In the primary and sensitivity models, these variables were mean-centred before entry into the mixed models, thereby allowing the intercept and lower-order coefficients to be interpreted at sample-mean levels of learning approach.

Topic selection and teaching procedure

Eight physiology topics were selected through a three-round Delphi process. The panel comprised all six full-time faculty members in the Department of Medical Physiology. An initial pool of 25 candidate topics was drawn from the national Bachelor of Medicine, Bachelor of Surgery (MBBS) physiology curriculum and rated for clinical relevance on a five-point Likert scale. This process followed a modified Delphi approach in which ratings were submitted anonymously. After each round, panellists received a summary of the group's median and interquartile range for every topic and were invited to revise their ratings in light of the group distribution. Consensus was predefined as a median score of at least four and an interquartile range of one or less. All six panellists participated in all three rounds. The topics, in the order taught, were Body Fluid Compartments and Oedema, Erythropoiesis and its Regulation, Neuromuscular Junction and Excitation-Contraction Coupling, Blood Pressure Regulation, Regulation of Respiration, Acid-Base Regulation, Sense of Hearing, and Autonomic Nervous System.

**Figure 1:**
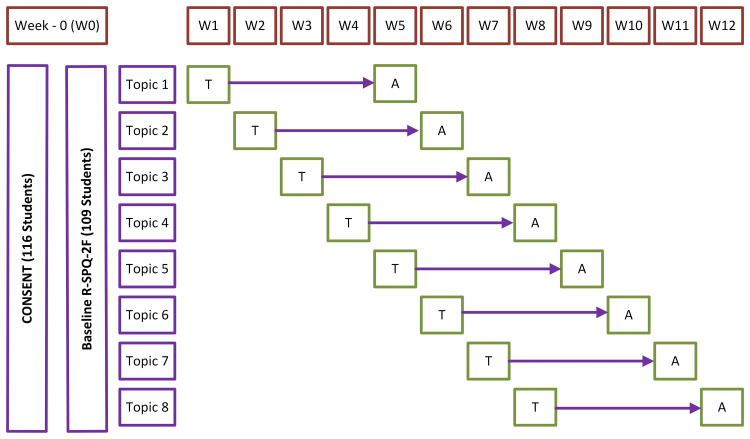
Study timeline and assessment design Out of the 150 eligible first-year medical students, 116 provided written informed consent, and 109 completed the baseline Revised Two-Factor Study Process Questionnaire (R-SPQ-2F) and were included in the complete-case analysis. Eight physiology topics were then taught sequentially during April and May 2025, with one topic delivered per week. In the figure, ‘W’ denotes study week, ‘T’ denotes the teaching session for a given topic, and ‘A’ denotes the corresponding assessment session, conducted approximately four weeks later on a rolling basis during May and June 2025

Following baseline R-SPQ-2F administration, two faculty members (four topics each) taught the eight topics using a traditional lecture-based format in April and May 2025 in face-to-face classroom sessions for the full cohort, typically on Mondays. Standardised lesson plans were used to ensure consistent duration, content coverage, and alignment with National Curriculum Outcomes across all topics and teachers. Assessments were conducted on a rolling basis in May and June 2025, with each topic assessed individually approximately four weeks after its teaching session, typically on a Wednesday, such that teaching and assessment overlapped from approximately week five onwards. All assessments were completed within three months of baseline R-SPQ-2F administration. These eight longitudinal formative tests formed part of the programme's broader year-long programmatic assessment framework, within which students undertook multiple formative assessments across the academic year. The overall study timeline and sequencing of teaching and assessment are shown (Figure 1).

For a given topic, both AFs were completed in the same sitting under examination conditions. The administration order of the two formats was fixed within each topic, with DAs administered first in four topics and MCQAs first in the remaining four. This design reduces but does not eliminate the possibility of systematic order effects; the topic was retained as a fixed effect to account for topic-related and sequencing differences.

Assessment design and scoring

For each topic, students completed two AFs: a descriptive assessment (DA) and a multiple-choice question assessment (MCQA). The principal investigator first generated a construct-matching matrix in which DA and MCQA items for each topic were drawn from the institution's existing summative assessment bank and mapped to the same curricular content, intended constructs, and revised Bloom's taxonomy level. This preliminary matrix was then submitted to the same three-round modified Delphi process described above, with the same panel, feedback procedure, and consensus criteria. During this process, panellists independently rated the construct and cognitive-level alignment of each item pair; where disagreements arose, items were reclassified or replaced until all final DA-MCQA pairs were confirmed as matched on both construct and Bloom's level. This design reduced the likelihood that any observed format differences in associations with learning approach were driven by systematic differences in the intended cognitive level of the item sets. No panellist was involved in marking the assessments.

Each DA followed a standardised examination question format familiar to students, comprising two four-mark short-answer questions and one two-mark very short-answer question; these mark allocations reflected usual examination practice and signalled the expected depth and scope of response, giving a total of 10 marks per topic. All DA scripts were marked by a single assessor using an analytic rubric with model answers drawn from the institution's existing assessment bank. The rubric allocated marks against specified criteria for each question (e.g., accuracy, completeness, reasoning), ensuring that scoring reflected predefined standards rather than marker judgement alone. To provide post hoc evidence on the consistency of DA scoring, two topics (Topic 2 and Topic 8) were selected at random, and all 218 scripts for these topics (approximately 25% of the total 872 DA scripts) were independently re-marked by a second faculty member using the same analytic rubric and model answers. Neither marker had access to students' learning-approach scores, and all scripts were marked using anonymised identifiers; the second marker was additionally blind to the original scores. Inter-rater reliability was evaluated using a two-way mixed-effects, single-measures, absolute-agreement intraclass correlation coefficient (ICC).

Each MCQA comprised 10 multiple-choice questions with four response options and one correct answer per item. Each correct answer scored one mark, with no negative marking, giving a total of 10 marks per topic. All MCQA scripts were scored by a postgraduate student using a predetermined answer key; as each item had a single correct response, scoring was objective. No prospective double-marking or formal moderation was undertaken for either format; however, a post hoc inter-rater reliability audit was conducted on a subset of DA scripts, as described above.

Analytic dataset and missing data

The primary analyses were conducted on a complete-case dataset. Out of the 116 consented students, 12 were absent on the day of baseline R-SPQ-2F administration. A single follow-up contact was made the following day, after which five responded; no further reminders were sent to avoid any perception of coercion in a student-participant context. The remaining seven non-respondents were excluded from analysis, as the R-SPQ-2F was required to derive the deep- and surface-learning covariates. Non-return appeared to reflect logistical non-response rather than any characteristic related to learning approach or academic performance. Because demographic data were not collected for excluded students, a formal comparison between included and excluded participants was not possible; this is acknowledged as a limitation. Among the 109 included students, complete data were available for all 16 assessment scores (eight topics × two formats) and both learning-approach subscales, yielding a long-format dataset of 1,744 observations. Because missingness was confined to missing baseline questionnaire data rather than intermittent missing outcome data within the repeated-measures dataset, no imputation was performed. Demographic characteristics of the analytic cohort and overall cohort were summarised descriptively to characterise the analysed sample.

Statistical analysis

All analyses were performed in IBM SPSS Statistics version 31.0 (IBM Corp., Armonk, NY), and the simulation-based analysis of achieved statistical power was conducted in R (version 4.5.3) using the simr, lme4, and lmerTest packages. Tests were two-tailed, and p < 0.05 was considered statistically significant. Ninety-five per cent confidence intervals (95% CI) were reported where appropriate. Two model-specification sensitivity analyses, described below, examined the robustness of the primary findings to alternative random-effects structures and learning-approach parameterisations, respectively. A further analysis, also described below, used simulation to assess whether the achieved sample provided adequate statistical power to detect pre-specified benchmark interaction effects.

Preliminary Analyses

Before fitting the mixed model, deep and surface learning scores were summarised descriptively using the mean, standard deviation (SD), minimum, and maximum values. Their association was examined using Pearson’s correlation coefficient with a 95% bootstrap percentile confidence interval (1,000 resamples). To assess whether both variables could be entered simultaneously in the same model without problematic collinearity, tolerance and variance inflation factor (VIF) values were examined. These checks were undertaken because the two learning-approach dimensions are conceptually related and, if highly correlated, could yield unstable coefficient estimates. Inter-rater reliability for the post hoc DA re-marking audit was assessed using a two-way mixed-effects, single-measures, absolute-agreement intraclass correlation coefficient (ICC), with 95% confidence intervals.

Primary Analysis

The primary inferential analysis used a linear mixed-effects model (LMM) to test whether the association between learning approach and assessment performance scores (marks) differed by AF. Marks were modelled as the dependent variable. Fixed effects were AF, topic, mean-centred deep learning score, mean-centred surface learning score, and the interactions between AF and each learning-approach variable. Deep and surface learning scores were mean-centred to improve interpretability of the intercept and lower-order coefficients, such that these estimates represented effects at the sample-mean level of the learning-approach variables. The topic was included as a fixed effect because the analysis concerned the eight specific prespecified physiology topics used in this study. The random-effects structure comprised a student-level random intercept to account for repeated observations within students. Models were estimated using restricted maximum likelihood (REML), with Satterthwaite approximation for denominator degrees of freedom. Although marks were bounded from 0 to 10, a Gaussian linear mixed model was considered appropriate because the primary aim was to estimate conditional mean differences rather than to model outcome probabilities, and because residual diagnostics did not indicate serious violations of normality or homoscedasticity assumptions. The primary random-effects structure used a variance-components specification, with student-level random intercept and residual variances estimated separately.

The primary model can be represented as:

$\mathrm{Marks}_{ij} = \beta_0 + \beta_1(\mathrm{Format}_j) + \beta_{2\mathrm{-}8}(\mathrm{Topic}_j) + \beta_9(\mathrm{Deep}_i) + \beta_{10}(\mathrm{Surface}_i)$

$+ \beta_{11}(\mathrm{Format}_j \times \mathrm{Deep}_i) + \beta_{12}(\mathrm{Format}_j \times \mathrm{Surface}_i) + u_{0i} + \varepsilon_{ij}$

where *i* indexes students and *j* indexes repeated observations within students. The term *u*_0*i*_ is a student-level random intercept capturing stable between-student differences in overall performance, assumed *u*_0*i*_ ~ N(0, σ^2^*_u_*), and *ε_ij_* is the observation-level residual, assumed εᵢⱼ ~ N(0, σ^2^*_e_*), with independence between the two components. In the primary parameterisation, MCQA was the reference category for AF. Accordingly, the lower-order coefficients for deep and surface learning represent their slopes within MCQAs, and the interaction terms represent the additional change in slope for DAs. To aid interpretation and avoid presenting duplicate re-parameterisations of the same model, simple slopes for deep and surface learning within each AF were estimated from this single fitted model using linear contrasts.

Model fit was summarised using the -2 restricted log likelihood, Akaike's Information Criterion (AIC), and Schwarz's Bayesian Criterion (BIC). Nakagawa-Schielzeth marginal and conditional pseudo-R^2^ values and intraclass correlation coefficients were also reported to characterise the variance explained and the degree of clustering [16]. To assess robustness to the simplifying random-intercept specification of the primary model, an additional sensitivity analysis was conducted in which AF was also allowed to vary randomly across students. Primary inference focused on the two format-by-learning-approach interaction terms. Format-specific associations were summarised using model-based simple slopes (linear contrasts) with 95% confidence intervals. The random-intercept specification was prespecified as the primary model on grounds of parsimony, with the random-slope sensitivity model, in which AF was allowed to vary across students, serving as a planned sensitivity analysis to evaluate robustness to a more flexible within-student specification, as described below.

Interaction Plots

For visualisation of the primary findings, model-based interaction plots were generated from the fitted primary linear mixed-effects model using fixed-effects predictions. Separate panels were produced for deep and surface learning by varying one mean-centred learning-approach predictor across its observed range while holding the other at its sample mean, equivalent to 0 after mean-centring. Predicted values were averaged across the eight topic levels. This ensured that the plotted lines represented the adjusted model-based simple slopes for MCQAs and DAs rather than unadjusted raw data patterns.

Model Diagnostics

Predicted values and residuals were saved from the fitted model and examined to assess model assumptions. Diagnostic procedures included a histogram of residuals with normal curve overlay, a scatterplot of predicted values against residuals, boxplots, stem-and-leaf plots, and normal probability plots. Descriptive summaries of the residuals, including skewness and kurtosis, were inspected, alongside Kolmogorov-Smirnov and Shapiro-Wilk tests of normality. Boxplots were also used to screen for extreme residual values that might indicate unusual observations. These diagnostics were used to evaluate the plausibility of normality and homoscedasticity assumptions, while recognising that formal normality tests are highly sensitive in large samples.

Simulation-Based Assessment of Achieved Sample Sufficiency

To assess the sensitivity of the achieved study design to detect modest format-dependent interaction effects, a simulation-based design sensitivity analysis was undertaken in R using the fitted primary mixed-effects model. Power was estimated for the two primary assessment-format-by-learning-approach interaction terms using 1,000 simulations. To avoid relying solely on an observed-power calculation based directly on the fitted coefficients, conservative interaction values of 0.03 and −0.03 were specified for the assessment-format-by-deep-learning and assessment-format-by-surface-learning terms, respectively. These values were selected as modest benchmark effects because they represent approximately 59% of the observed deep-learning interaction coefficient (0.051) and 53% of the observed surface-learning interaction coefficient (−0.057), thus providing a more conservative test of the achieved design than a direct observed-power restatement. The simulation retained the observed repeated-measures structure, including the number of students, the number of observations per student, and the estimated variance structure of the primary model.

Sensitivity Analysis: Random Slope for Assessment Format

To examine whether inference on the key assessment-format-by-learning-approach interactions was robust to a more flexible within-student random-effects structure, a planned sensitivity analysis was undertaken in which the AF effect was additionally allowed to vary across students. This model retained the same fixed-effects specification as the primary model, namely AF, topic, mean-centred deep-learning score, mean-centred surface-learning score, and the interactions between AF and each learning-approach variable. The random-effects structure included a student-level random intercept and a random slope for AF, specified with an unstructured covariance matrix. As in the primary model, MCQA served as the reference category; thus, the lower-order coefficients for deep learning and surface learning represented their slopes within MCQAs, whereas the corresponding interaction terms represented the difference in slope for DAs relative to MCQAs. Simple slopes within each format were estimated using linear contrasts, as in the primary model. The model was estimated using restricted maximum likelihood, with Satterthwaite approximation for denominator degrees of freedom.

Cross-Topic Consistency of Performance

As a supplementary psychometric analysis, Cronbach’s alpha was calculated separately for the eight topic-level totals within each AF. Because these totals represented performance across different curricular topics rather than items within a single test, alpha was interpreted cautiously as an index of cross-topic score consistency within each format rather than conventional item-level internal consistency. For completeness, inter-topic correlations, corrected topic-total correlations, and alpha-if-topic-deleted statistics were also examined to assess whether any single topic disproportionately influenced the cross-topic consistency estimate. This analysis was descriptive and supplementary, and was not used to infer unidimensionality of either AF.

Sensitivity Analysis: Relative Learning Orientation

Given that deep and surface learning approaches are conceptually related and may show a negative empirical association, a sensitivity analysis was undertaken using a single composite index of relative learning orientation, defined as the deep-approach score minus the surface-approach score. This variable was mean-centred before analysis. An otherwise analogous linear mixed-effects model was then fitted with AF, topic, mean-centred Deep-minus-Surface score, and the interaction between AF and Deep-minus-Surface as fixed effects, together with a student-level random intercept. As in the primary model, MCQA served as the reference category, and simple slopes within MCQAs and DAs were estimated using linear contrasts. This sensitivity analysis examined whether the substantive conclusions were robust to a more parsimonious representation of the learning approach. Because this sensitivity model differed from the primary model in fixed-effects specification and both models were estimated using restricted maximum likelihood, information criteria were presented descriptively only and were not used for formal model comparison or to infer model superiority.

## Results

Participants

Of the 116 students who consented to participate, seven were excluded because they did not complete the R-SPQ-2F questionnaire, leaving a complete-case dataset of 109 students with data across all 16 assessment scores and both learning-approach subscales. As each student contributed scores for eight physiology topics in two AFs, the long-format dataset comprised 1,744 observations. The distribution of admission categories in the analytic cohort was similar to that of the overall class (Table 1). Because demographic data were not available for non-respondents to the baseline questionnaire, representativeness with respect to age and gender could not be formally assessed.

**Table 1:** Student characteristics of the overall cohort and analytic cohort Category A = government quota seats; Category B = management quota seats; Category C = non-resident quota seats. These categories reflect distinct admission pathways that differ in entrance examination ranking thresholds. Age and gender data were available only for the analytic cohort (n = 109); these variables could not be obtained for non-respondents. Gender was self-reported. Female and male percentages are calculated within each admission category for the analytic cohort SD: standard deviation

Characteristic	Category	Overall cohort, n (%)	Analytic cohort			
			Study cohort, n (%)	Age, years, mean ± SD	Female, n (%)	Male, n (%)
Category-wise composition	A	90 (60.0)	67 (61.5)	20.55 ± 1.42	47 (70.1)	20 (29.9)
	B	37 (24.7)	27 (24.8)	20.85 ± 1.51	17 (62.0)	10 (37.0)
	C	23 (15.3)	15 (13.8)	19.87 ± 0.92	9 (60.0)	6 (40.0)
Total		150 (100)	109 (100.0)	20.53 ± 1.41	73 (67.0)	36 (33.0)

Preliminary analyses

Before fitting the primary model, baseline deep and surface learning scores were summarised to describe their range and variability, and to assess whether the two learning-approach variables were sufficiently distinct to be entered together as predictors. On the R-SPQ-2F subscales, deep approach scores ranged from 12 to 47 (mean = 31.62, SD = 7.88) and surface approach scores ranged from 10 to 50 (mean = 22.90, SD = 8.74). The two scores were modestly but significantly negatively correlated (r = −0.268, 95% CI −0.47 to −0.03, p = 0.005, n = 109), indicating that the constructs overlap but are not redundant.

Internal consistency of the R-SPQ-2F subscales in the present cohort was acceptable. Cronbach’s alpha was 0.720 for the deep-approach subscale and 0.770 for the surface-approach subscale, based on the 10 items within each subscale (n = 109). These values indicate adequate reliability for use as continuous predictors, although any associations with marks may have been slightly underestimated because the subscales were not perfectly reliable.

Collinearity diagnostics confirmed that deep and surface scores could be entered together without inflating standard errors (SEs). Tolerance was 0.928 for both predictors, and the variance inflation factor (VIF) was 1.078, well below conventional thresholds for concern. These checks supported the simultaneous inclusion of both predictors in the primary mixed-effects model. Across all topics and students (872 observations per format), MCQA marks ranged from 0 to 10 (mean = 5.45, SD = 2.30) and DA marks ranged from 0 to 9.5 (mean = 7.11, SD = 1.83). DA marks were, on average, higher but showed less variability than MCQA marks, indicating that the weaker MCQA slopes in subsequent models were not attributable to restricted variance in MCQA scores.

In the post hoc inter-rater reliability audit, the two-way mixed-effects, single-measures, absolute-agreement ICC across the 218 re-marked DA scripts (Topics 2 and 8) was 0.873 (95% CI 0.838-0.901, F(217, 217) = 14.77, p < 0.001), indicating strong inter-rater agreement. This provides reassurance that DA scoring using the analytic rubric was reproducible across independent markers and reduces concern that the format-dependent associations observed in subsequent models were substantially driven by marker inconsistency.

Primary analysis

Model Fit and Variance Components

The primary LMM described in Methods was fitted to all 1,744 observations from 109 students. Fit statistics were as follows: −2 restricted log likelihood = 7,311.0, AIC = 7,315.0, and BIC = 7,325.9. The fixed effects accounted for approximately 17% of the variance in marks (Nakagawa-Schielzeth marginal pseudo-R^2^ = 0.167); including the random intercept increased the conditional pseudo-R^2^ to approximately 31% (0.313).

The estimated residual variance was 3.460 (SE = 0.121, 95% CI 3.230-3.706) and the random intercept variance was 0.737 (SE = 0.131, 95% CI 0.520-1.044). The adjusted intraclass correlation coefficient was 0.176, indicating that approximately 18% of the total variance in marks was attributable to stable between-student differences in overall performance.

Fixed Effects and Interaction Terms

At sample-mean values of deep and surface learning, AF was strongly associated with performance, F(1, 1625) = 346.45, p < 0.001. Adjusted marks were 1.658 points higher on DAs than on MCQAs (SE = 0.089, 95% CI 1.483-1.833). Topic effects were not statistically significant in this model, F(7, 1625) = 0.89, p = 0.515. The intercept represents the expected MCQA mark for the reference topic (Topic 8) at sample-mean values of both learning-approach scores. Given the small and non-significant topic coefficients, this value closely approximates the overall MCQA mean. The full set of fixed-effect estimates is presented (Table 2).

**Table 2:** Primary linear mixed-effects model: estimates of fixed effects DA is a descriptive assessment, and MCQA is a multiple-choice question assessment. MCQA is the reference category for the assessment format. Topic coefficients (seven dummy variables) were included in the model but are omitted for brevity; none was individually significant. The rows labelled “Deep-learning slope in MCQA (reference format)” and “Surface-learning slope in MCQA (reference format)” give the simple slopes of the respective learning-approach variables within the MCQA reference category. The interaction rows give the additional change in those slopes for DAs relative to MCQAs. Thus, the DA slopes are obtained by summing the MCQA slope and the corresponding interaction term. Model fit: −2 restricted log likelihood = 7,311.0; AIC = 7,315.0; BIC = 7,325.9; marginal R^2^ = 0.167; conditional R^2^ = 0.313. Residual variance = 3.460; random intercept variance (student) = 0.737; adjusted ICC = 0.176 SE: standard error; df: degrees of freedom; CI: confidence interval; AIC: Akaike's Information Criterion; BIC: Schwarz's Bayesian Criterion; ICC: intraclass correlation coefficient

Parameter	Estimate (β)	SE	df	t	p	95% CI
Intercept	5.451	0.157	691.40	34.74	<0.001	5.143, 5.759
DA vs MCQA	1.658	0.089	1625.00	18.61	<0.001	1.483, 1.833
Deep learning slope in MCQA (reference format)	−0.019	0.014	159.03	−1.42	0.157	−0.047, 0.008
Surface learning slope in MCQA (reference format)	0.019	0.012	159.03	1.54	0.125	−0.005, 0.043
Difference in deep-learning slope: DA vs MCQA	0.051	0.012	1625.00	4.34	<0.001	0.028, 0.074
Difference in surface-learning slope: DA vs MCQA	−0.057	0.011	1625.00	−5.34	<0.001	−0.078, −0.036

Within MCQAs, neither the deep-approach nor the surface-approach score showed clear evidence of association with marks. In contrast, both predictors interacted significantly with AF, indicating that their associations with marks differed between MCQAs and DAs. The interaction between AF and deep learning was significant, F(1, 1625) = 18.81, p < 0.001, as was the interaction between AF and surface learning, F(1, 1625) = 28.49, p < 0.001. Format-specific simple slopes are reported in the following section.

Simple Slopes by Assessment Format

To clarify the nature of the interactions, simple slopes were estimated for the effect of each learning-approach variable within each AF separately.

Within MCQAs, neither learning-approach variable was significantly associated with marks. Deep learning showed no significant relationship with MCQA performance (β = −0.019, SE = 0.014, t = −1.42, df = 159.03, p = 0.157, 95% CI −0.047 to 0.008), and surface learning was likewise unrelated (β = 0.019, SE = 0.012, t = 1.54, df = 159.03, p = 0.125, 95% CI −0.005 to 0.043).

**Figure 2:**
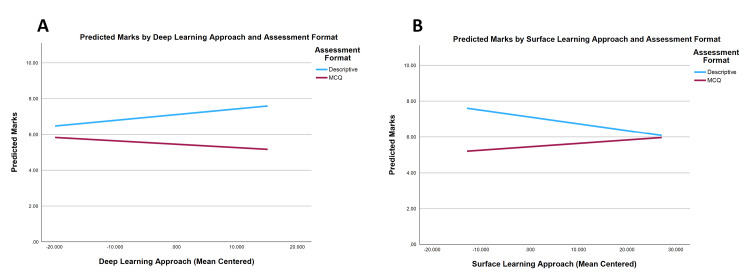
Adjusted associations between learning approach and marks by assessment format Adjusted associations between learning approach and marks by assessment format from the primary linear mixed-effects model. (A) Predicted marks (0–10) across the observed range of mean-centred deep learning scores (R-SPQ-2F points), with surface learning held at 0. (B) Predicted marks (0–10) across the observed range of mean-centred surface learning scores (R-SPQ-2F points), with deep learning held at 0. In both panels, predicted values were derived from the fixed effects of the fitted primary model and averaged across topic effects. Lines show fixed-effect predictions; uncertainty bands are not displayed. Descriptive-assessment marks increased with deep learning and decreased with surface learning, whereas the evidence for associations with either learning-approach dimension within MCQs was weaker, with confidence intervals compatible with small positive or negative slopes R-SPQ-2F: Revised Two-Factor Study Process Questionnaire; MCQs: multiple-choice question assessments

A markedly different pattern emerged for DAs. Higher deep-learning scores were associated with higher marks (β = 0.032, SE = 0.014, t = 2.31, df = 159.03, p = 0.022, 95% CI 0.005-0.059), whereas higher surface-learning scores were associated with lower marks (β = −0.038, SE = 0.012, t = −3.05, df = 159.03, p = 0.003, 95% CI −0.062 to −0.013). The difference between the deep-learning slope in DAs(+0.032) and in MCQAs (−0.019) was 0.051 (p < 0.001). This confirms that the format-dependent pattern reflects a genuine change in the direction and magnitude of the association, not merely a difference in significance thresholds. DAs showed clearer evidence of association with learning-approach scores than MCQAs in this cohort, whereas the evidence for associations within MCQAs was weaker, with confidence intervals compatible with small positive or negative slopes. These adjusted format-specific associations are illustrated (Figures 2A, 2B), with model-predicted marks plotted for each learning-approach variable across its observed range while holding the other mean-centred learning-approach variable at zero.

To aid interpretation, slopes can be translated into the expected mark difference associated with a one-standard-deviation change in the predictor. For DAs, a one-SD increase in deep learning was associated with an increase of approximately 0.25 marks (slope 0.032 × SD 7.88) (Figure 2A) and a one-SD increase in surface learning with a decrease of approximately 0.33 marks (slope −0.038 × SD 8.74) (Figure 2B). For MCQAs, the corresponding values were approximately −0.15 marks per SD of deep learning (slope −0.019 × SD 7.88) and +0.17 marks per SD of surface learning (slope 0.019 × SD 8.74), neither of which was statistically significant. The simple slopes are summarised (Table 3).

**Table 3:** Simple slopes: association between learning approach and marks within each assessment format DA is a descriptive assessment, and MCQA is a multiple-choice question assessment. Simple slopes were estimated from the primary linear mixed-effects model (Table 2) using linear contrasts. MCQA is the reference category for the assessment format. The DA slopes are obtained by summing the MCQA slope and the corresponding interaction term from Table 2 SE: standard error; df: degrees of freedom; CI: confidence interval

Assessment format	Predictor	Estimate (β)	SE	df	t	p	95% CI
MCQA	Deep learning	−0.019	0.014	159.03	−1.42	0.157	−0.047, 0.008
MCQA	Surface learning	0.019	0.012	159.03	1.54	0.125	−0.005, 0.043
DA	Deep learning	0.032	0.014	159.03	2.31	0.022	0.005, 0.059
DA	Surface learning	−0.038	0.012	159.03	−3.05	0.003	−0.062, −0.013

Model Diagnostics

Residual diagnostics did not indicate serious violations of model assumptions. The distribution of residuals was approximately symmetric, with mild negative skew (skewness = −0.23, SE = 0.06) and slightly platykurtic kurtosis (kurtosis = −0.45, SE = 0.12). Although the Kolmogorov-Smirnov (D = 0.055, p < 0.001) and Shapiro-Wilk tests (W = 0.990, p < 0.001) were statistically significant, these tests are highly sensitive in large samples such as this one (N = 1,744). Visual inspection of the histogram and normal Q-Q plot supported approximate normality, with only minor tail departures. The residual-versus-predicted plot showed the expected banding for a discrete 0-10 marks outcome, but no obvious fanning or curvilinear pattern. Overall, the assumptions of approximate normality and homoscedasticity were considered adequately met.

Simulation-Based Assessment of Achieved Sample Sufficiency

In the simulation-based design sensitivity analysis of the primary mixed-effects model, the achieved study design provided 72.9% power (95% CI 70.03-75.63) to detect an assessment-format-by-deep-learning interaction coefficient of 0.03 marks per one-point increase in deep-learning score, and 81.6% power (95% CI 79.06-83.96) to detect an assessment-format-by-surface-learning interaction coefficient of −0.03 marks per one-point increase in surface-learning score. The value of 0.03 was used as a deliberately conservative benchmark, smaller than the observed interaction coefficients of 0.051 and −0.057, rather than as a post hoc power calculation based directly on the observed effects. These benchmark values represented approximately 59% and 53% of the corresponding observed interaction coefficients, respectively. The simulations therefore suggest that the achieved design had moderate-to-good sensitivity to detect modest format-dependent associations under the specified benchmarks.

Sensitivity analysis: random slope for assessment format

To assess robustness to the simplifying random-intercept structure of the primary model, a sensitivity analysis was conducted in which AF was additionally allowed to vary randomly across students. This model converged successfully. The estimated random intercept variance was 1.206 (SE = 0.215, 95% CI 0.851-1.710), the random slope variance for AF was 2.366 (SE = 0.423, 95% CI 1.666-3.360), and the intercept-slope covariance was -1.023 (SE = 0.252, 95% CI -1.516 to -0.529), indicating substantial between-student variability in the format effect. The residual variance was 2.842 (SE = 0.103, 95% CI 2.647-3.052). Despite this additional variability, the model yielded substantively similar conclusions. The interaction between AF and deep learning remained significant, F(1, 106.001) = 5.286, p = 0.023, as did the interaction between AF and surface learning, F(1, 106.001) = 8.008, p = 0.006, indicating that the principal inference of format-dependent associations between learning approach and marks was robust to this more flexible random-effects specification.

Simple slopes were directionally unchanged. Within MCQAs, neither deep learning (β = −0.019, SE = 0.016, t = −1.229, df = 106.004, p = 0.222, 95% CI −0.051 to 0.012) nor surface learning (β = 0.019, SE = 0.014, t = 1.335, df = 106.004, p = 0.185, 95% CI −0.009 to 0.047) was significantly associated with marks. Within DAs, the slope for surface learning remained significantly negative (β = −0.038, SE = 0.016, t = −2.402, df = 106.007, p = 0.018, 95% CI −0.069 to −0.007), whereas the evidence for the positive deep-learning slope within DAs was weaker and no longer reached conventional statistical significance (β = 0.032, SE = 0.017, t = 1.819, df = 106.007, p = 0.072, 95% CI −0.003 to 0.066).

Under the random-slopes specification, the format-by-deep interaction remained statistically significant, but the statistical evidence for the deep-learning simple slope within DAs became less precisely estimated and did not meet the conventional 0.05 threshold (p = 0.072). Conclusions about deep learning within DAs should therefore be framed as weaker evidence than conclusions about the interaction itself. The random-intercept model was retained as the primary analysis on grounds of parsimony; the random-slopes model is presented as a sensitivity check to assess the robustness of the primary findings to a more flexible within-student specification.

Cross-topic consistency of performance

As a descriptive supplement, we summarised cross-topic consistency of topic-level totals within each format, including alpha and inter-topic correlations, recognising that these are not conventional internal-consistency analyses. Because these totals came from different curricular topics rather than items within a single test, the resulting alpha values are best interpreted as indices of cross-topic performance consistency rather than conventional item-level internal-consistency estimates. High alpha values may therefore partly reflect stable between-student differences in general academic performance rather than homogeneity of the assessment itself.

With that caveat, descriptive-assessment totals showed high cross-topic consistency (α = 0.911), with inter-topic correlations ranging from 0.48 to 0.64. All corrected topic-total correlations exceeded 0.65, and removal of any single topic would have reduced alpha, indicating that each topic contributed to the overall pattern. MCQA totals showed lower and more variable cross-topic consistency (α = 0.671), with inter-topic correlations ranging from −0.024 to 0.40. Several MCQA topic totals had low corrected topic-total correlations, including Topic 5 (r = 0.23), and removal of individual topic totals would have had only modest effects on the overall alpha. Student performance was therefore more consistent across topics in DAs than in MCQAs (Table 4); however, because these totals span different topics, this pattern may reflect stable between-student differences rather than a single underlying skill.

**Table 4:** Consistency of performance across topic-level totals within each assessment format Alpha values are interpreted here as indices of cross-topic consistency within each format, not as conventional item-level internal-consistency estimates for a single test MCQ: multiple-choice question

Metric	Descriptive assessment	MCQ assessment
Number of topic totals	8	8
Cronbach’s alpha	0.911	0.671
Inter-topic correlation range	0.48 – 0.64	−0.024 – 0.40
Corrected item–total correlation range	0.66 – 0.76	0.23 – 0.50

Sensitivity analysis: relative learning orientation

As a sensitivity analysis, a single composite measure of relative learning orientation, defined as deep minus surface learning score, was examined to determine whether it yielded substantively similar findings. This alternative specification examined whether a more deep-oriented relative profile was associated with differential performance across formats, while providing a more parsimonious representation of the learning approach. The Deep-minus-Surface variable was derived from the baseline R-SPQ-2F and was therefore constant within each student. It was mean-centred before entry into the model.

The sensitivity model followed the same general structure as the primary model but replaced the two separate learning-approach covariates with the single Deep-minus-Surface score and its interaction with AF. Because this sensitivity model differed from the primary model in fixed-effects specification and both models were estimated using restricted maximum likelihood, information criteria are presented descriptively only and were not used to infer model superiority. Fit statistics were as follows: −2 restricted log likelihood = 7,298.8, AIC = 7,302.8, and BIC = 7,313.7. The marginal pseudo-R² was 0.167, and the conditional pseudo-R² was 0.312, closely mirroring the primary model. The residual variance was 3.458, and the random intercept variance was 0.728; the adjusted intraclass correlation coefficient was 0.174.

At the sample-mean Deep-minus-Surface score, AF was again strongly associated with performance, F(1, 1626) = 346.75, p < 0.001, and topic again showed no significant effect, F(7, 1626) = 0.89, p = 0.515. The interaction between AF and the Deep-minus-Surface score was highly significant, F(1, 1626) = 64.29, p < 0.001, indicating that the relationship between relative learning orientation and marks differed clearly by AF. The principal fixed-effect estimates are presented (Table 5).

**Table 5:** Sensitivity model: estimates of fixed effects (Deep-minus-Surface) DA is a descriptive assessment, and MCQA is a multiple-choice question assessment. MCQA is the reference category. Topic coefficients were included but are omitted for brevity. The row labelled “Deep-minus-Surface slope in MCQA (reference format)” gives the slope of Deep-minus-Surface within the MCQA reference category; the interaction row gives the additional change in slope for DAs. Thus, the DA slope is obtained by summing these two coefficients. Fit statistics are reported descriptively only because this sensitivity model differed from the primary model in fixed-effects specification, and both models were estimated using restricted maximum likelihood. Model fit: −2 restricted log likelihood = 7,298.8; AIC = 7,302.8; BIC = 7,313.7; marginal R2 = 0.167; conditional R2 = 0.312. Residual variance = 3.458; random intercept variance = 0.728; adjusted ICC = 0.174 SE: standard error; df: degrees of freedom; CI: confidence interval; AIC: Akaike's Information Criterion; BIC: Schwarz's Bayesian Criterion; ICC: intraclass correlation coefficient

Parameter	Estimate (β)	SE	df	t	p	95% CI
Intercept	5.451	0.157	702.37	34.81	<0.001	5.143, 5.758
Descriptive vs. MCQA	1.658	0.089	1626.00	18.62	<0.001	1.484, 1.833
Deep-minus-Surface slope in MCQA (reference format)	−0.019	0.008	161.01	−2.46	0.015	−0.035, −0.004
Difference in Deep-minus-Surface slope: DA vs MCQA	0.054	0.007	1626.00	8.02	<0.001	0.041, 0.067

Simple slopes revealed that within MCQAs, a more deep-oriented relative profile was associated with slightly lower marks (β = −0.019, SE = 0.008, t = −2.46, df = 161.01, p = 0.015, 95% CI −0.035 to −0.004). This composite captures relative orientation rather than either learning-approach dimension alone, and this MCQA finding should therefore be interpreted cautiously. In contrast, within DAs, a more deep-oriented relative profile was associated with higher marks (β = 0.035, SE = 0.008, t = 4.46, df = 161.01, p < 0.001, 95% CI 0.019-0.050). Thus, the association between relative learning orientation and marks differed in direction across formats, being negative in MCQAs and positive in DAs. This sensitivity analysis therefore supported the primary finding that the association between learning approach and marks differed by AF. Format-specific simple slopes are summarised (Table 6).

**Table 6:** Simple slopes from the sensitivity model: Deep-minus-Surface within each format DA is a descriptive assessment, and MCQA is a multiple-choice question assessment. Simple slopes were estimated from the sensitivity model (Table 5) using linear contrasts. MCQA is the reference category for the assessment format. The DA slope is obtained by summing the MCQA slope and the interaction term from Table 5 SE: standard error; df: degrees of freedom; CI: confidence interval

Assessment format	Estimate (β)	SE	df	t	p	95% CI
MCQA	−0.019	0.008	161.01	−2.46	0.015	−0.035, −0.004
DA	0.035	0.008	161.01	4.46	<0.001	0.019, 0.050

## Discussion

Principal findings

This study provides evidence that the association between learning approach and assessment performance (marks) differed by AF. In the primary LMM, DAs showed a modest positive association with deep learning and a modest negative association with surface learning, whereas MCQAs showed little evidence of association with either learning-approach dimension. The key finding was therefore not that MCQAs clearly rewarded surface learning, but that DAs showed clearer differentiation by learning approach than MCQAs in this cohort. This interpretation is supported by the significant AF-by-deep-learning and AF-by-surface-learning interaction terms. When expressed in standardised units, the DA effects represented shifts of roughly 0.13 to 0.18 residual standard deviations, and this format-dependent pattern persisted when slopes were scaled per one standard deviation of the predictor, reducing concern that the interaction is attributable to differences in score scale or variance across formats.

However, the evidence for the positive deep-learning association in DAs should be interpreted cautiously, as its statistical strength was attenuated and no longer conventionally significant in the random-slopes sensitivity analysis; by contrast, the negative surface-learning association in DAs was robust across both model specifications. Accordingly, the most robust conclusion, in this cohort, is that AF modified the association between learning approach and performance, with stronger evidence of differentiation in DAs, particularly for poorer DA performance at higher surface-learning scores. A complementary sensitivity model using a Deep-minus-Surface composite yielded the same overall pattern of AF-dependent association, supporting the robustness of the central interaction finding across alternative parameterisations of the learning approach. Notably, the composite model revealed a significant negative MCQA slope that was absent in the primary two-covariate model; this shift should be interpreted cautiously, because the composite conflates deep and surface dimensions into a single index of relative orientation, and a negative MCQA slope may therefore reflect the combined influence of both dimensions rather than a pure deep-learning effect.

Taken together, these findings directly address the study aim by showing that AF modified the extent to which baseline learning-approach scores were reflected in marks, rather than demonstrating a uniform main effect of learning approach across written assessments. However, this greater covariation in DAs could partly reflect differences in writing proficiency or format-specific reliability rather than cognitive engagement alone, although the strong inter-rater agreement observed in the post hoc reliability audit (ICC = 0.873) reduces concern that rater inconsistency substantially contributed to this pattern.

As a descriptive psychometric supplement, cross-topic score consistency was higher for DA totals than for MCQA totals in this cohort. However, these values should be interpreted cautiously because they were calculated across totals from different curricular topics rather than across items within a single test. The higher alpha for DAs, therefore, cannot be taken as evidence that they measured a single construct; as noted in the Results, this greater consistency may partly reflect stable between-student differences, such as general academic ability or writing proficiency, rather than a format-specific skill. Likewise, the lower reliability values in MCQAs do not demonstrate that they only assessed recognition or factual recall. Rather, these supplementary findings indicate that performance was more stable across topics in the DA format within this cohort, while the primary inferential evidence for differential educational consequences remains the significant AF-by-learning-approach interactions.

Association of assessment format and learning approach

Our findings align with prior theoretical perspectives and empirical research in suggesting that constructed-response and selected-response AFs may differ in how strongly assessment performance reflects variation in learning approach. In the present study, DAs showed clearer differentiation by learning approach than MCQAs. However, the cognitive processes underlying this pattern were not directly observed, and well-designed MCQs can also evoke higher-order processing [17]. Any explanation that attributes the observed format-dependent pattern to specific cognitive processes should therefore be treated as inferential rather than directly demonstrated.

Biggs and Tang note that students adopting a predominantly surface learning approach may prefer MCQAs over DAs [5]. Supporting this, Amin et al. found that medical students who favoured MCQs highlighted the possibility of obtaining the correct answer even when unfamiliar with the content, and valued the presence of both question and answer as a stimulus for memory. Conversely, those who preferred essay questions cited the opportunity to think critically and engage more meaningfully with the learning material [18]. More recent evidence from dental students by Arooj et al. demonstrated a strong positive correlation between deep learning and performance in short essay questions, but only a very weak correlation between deep learning and performance in MCQs. Importantly, they also reported the inverse: surface learning was positively associated with MCQ performance but detrimental to DA outcomes [15].

Early work by Scouller reported that MCQs were associated with surface learning approaches, whereas essay-based tasks were associated with deeper conceptual processing [14]. Cilliers et al. also note that in medical education, constructed-response and open-ended assessments were more frequently associated with deep learning, whereas selected-response formats tended to be associated with surface, reproductive approaches [19]. Evidence from pharmacy education indicates that performance on DAs is associated with mastery-approach motivation, consistent with deep learning, while performance on MCQs is linked with avoidance-oriented motivation, a characteristic of surface strategies [20]. In a large cohort of Brazilian medical students, preference for MCQs was correlated with surface approaches like memorisation, and last-minute preparation [21].

Given this tendency, Biggs and Tang criticise MCQs for their limited capacity to assess higher-order learning outcomes [5]. Within a physiology context, Heber et al. showed that performance decreased markedly when tasks shifted from multiple-choice recognition (79% correct) to factual recall without options (73%), and further to open-ended descriptive questions requiring conceptual elaboration (26%) [22]. This pattern is consistent with the view that MCQAs may sometimes be completed with greater reliance on recognition and recall, whereas DAs more directly require response generation and elaboration.

Implications for assessment design

Despite the widespread criticism of MCQAs for testing superficial knowledge, it is important to acknowledge that well-constructed MCQs can assess higher-order skills [17]. Shaibah and van der Vleuten, in anatomy education, demonstrated strong correlations between MCQs and free-response items, even for higher-order tasks, concluding that MCQs can validly assess application and understanding if stimulus design is appropriate [23]. Indeed, Zaidi et al. demonstrated that MCQs targeting higher Bloom's taxonomy levels can require active retrieval and integration of concepts rather than simple recognition, suggesting that the cognitive demands of MCQs depend substantially on item construction [17]. The framing of MCQAs as purely recognition-based, therefore, oversimplifies the cognitive processes they can elicit. This highlights that the cognitive level measured depends more on item quality and construct validity than on the AF per se. Schuwirth and van der Vleuten further argue that assessment quality is determined not by AF alone but by the validity of assessment and item design and integration within a broader programme of assessment [9,13].

Empirical work supports this nuance. Palmer and Devitt found that over half of the modified essay questions in their study tested only factual recall, making them comparable to MCQs in that study, and concluded that carefully designed MCQs can effectively assess higher-order cognitive skills [24]. Similarly, Hift has argued that the poor inter-rater reliability of even well-constructed DAs limits their suitability for summative assessment, favouring instead well-designed MCQs that can test higher-order processes while maintaining objectivity [25]. Indeed, objectivity remains a key undisputed advantage of MCQs in high-stakes contexts such as postgraduate selection [26]. In our study context, both the DA and MCQA items were drawn from a summative bank where performance on summative assessment determines progression. Within our educational context, decisions on progression are informed by performance across diverse AFs, including DAs, MCQAs, practical skills assessments, and oral examinations. The complementarity of AFs helps ensure that progression decisions rest on sound assessment principles [9,13]. This approach aligns with the programmatic assessment framework, in which the value of each AF is judged by its contribution to an integrated system rather than in isolation, and in which the combination of formative and summative purposes within that system is a critical design consideration [9,13].

However, given our findings, a complete reliance on MCQs alone in high-stakes contexts, such as postgraduate selection [26], warrants caution. In the present study, DAs showed clearer differentiation by learning approach than MCQAs, whereas the MCQA slopes in the primary LMM were small and non-significant. Accordingly, our findings do not show that MCQAs inherently reward surface learning, but they do suggest that within this cohort and with the selected item sets, MCQAs provided less differentiation by learning approach than DAs. This emphasises the need to review assessment items carefully and ensure that they are designed to test deep knowledge.

While our study concentrated on written AFs (DA and MCQA), we acknowledge alternative AFs that foster deep learning. For example, Heber reported an increase from 26% to 47% in performance on conceptual knowledge tests after the introduction of oral examinations, which allowed students to elaborate and justify their reasoning [22]. Similarly, Delgado et al. found that students who adopted deep approaches expressed a preference for practical examinations [21].

Educational implications

Beyond decision-making for progression or licensure, it is important to recognise that assessment strongly influences learning. The value of formative assessment lies in its capacity to influence learning behaviours by providing insight through feedback and enabling periodic course correction before end-point summative assessment [19]. Hence, feedback is a crucial link through which assessments fulfil their role as ‘assessment for learning’ [9,13].

Clack and Dommett provide a useful caution against attributing the learning approach to AF alone. They reported that coursework essays were typically associated with deep approaches and MCQs with surface strategies, but this distinction disappeared when both formats were administered under identical examination conditions. They also observed that students with a greater deep learning approach actively sought detailed written feedback, whereas students with surface learning were more often satisfied with grades alone [27]. Hence, the educational effect of assessment depends not only on AF, but also on assessment conditions and students’ engagement with feedback [13,27]. In the present study, feedback quality, specificity, and learner uptake were not measured. Rather, we aimed to examine whether AFs differed in their capacity to differentiate performance according to more educationally desirable deep approaches and less desirable surface approaches. Although we did not evaluate feedback directly, an assessment that differentiates performance by learning approach may provide the diagnostic basis for more targeted feedback; whether that feedback subsequently modifies learning depends on its quality, timing, specificity, and uptake by learners.

**Figure 3:**
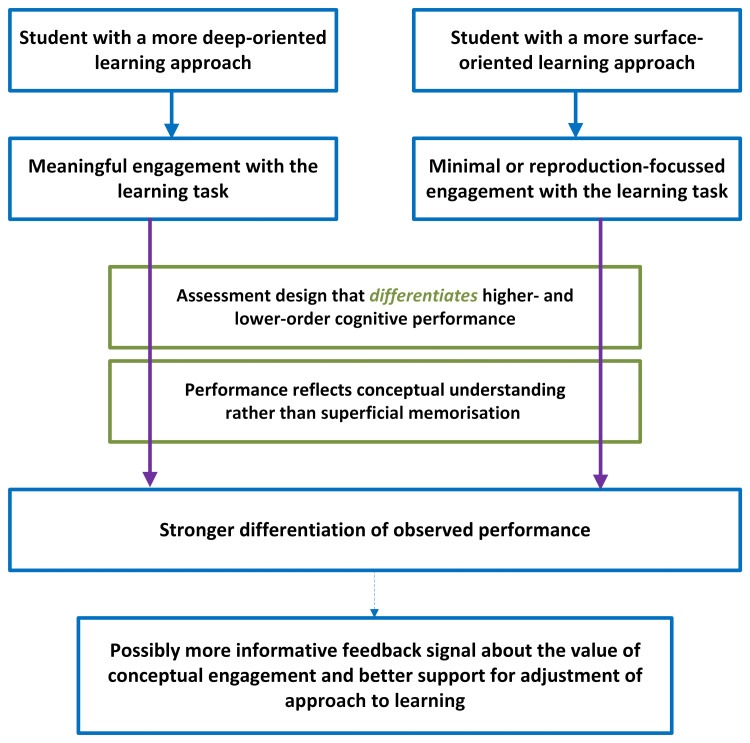
Conceptual model of assessment that differentiates learning outcomes A conceptual model in which an assessment that distinguishes higher- from lower-level cognitive outcomes is more likely to reward conceptual understanding than rote recall. Such differentiation may generate more informative feedback about the value of meaningful engagement and may better support students’ adjustment of study strategies

**Figure 4:**
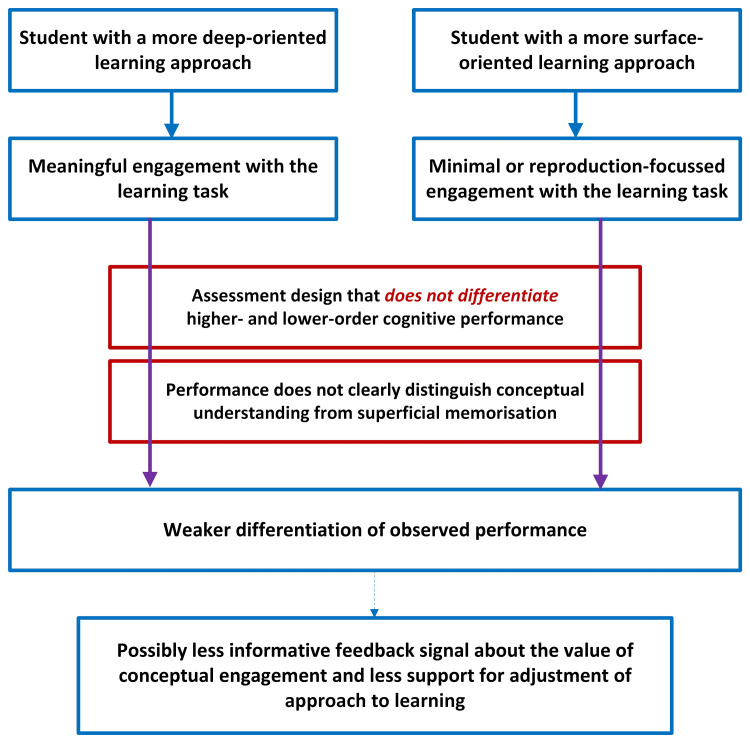
Conceptual model of assessment that does not differentiate learning approaches A conceptual model in which an assessment that does not clearly distinguish between deeper and more superficial engagement may yield similar outcomes across learners with different approaches, thereby offering less informative feedback about the value of conceptual understanding

These implications can be represented schematically. An assessment format that differentiates between higher- and lower-level cognitive outcomes may generate a more informative feedback signal regarding deeper versus more superficial engagement with learning (Figure 3). Conversely, an assessment format that does not clearly differentiate these outcomes may yield similar marks regardless of learning approach and therefore provide less informative feedback for guiding improvement (Figure 4).

This interpretation should not be taken to imply that AF alone determines learning approach or performance. Although assessment is widely recognised as an important influence on student engagement with learning tasks, the empirical evidence is not entirely consistent. A longitudinal study reported no significant association between learning approaches and performance across different AFs [28]. Similarly, a systematic review reported mixed findings for surface learning: among 49 included studies, 24 reported a negative association between surface learning and academic performance, 26 reported no significant association, and one reported a positive association; these counts were not mutually exclusive because some studies showed mixed findings [29]. These findings reinforce the view that assessment operates within a broader educational environment, in which student characteristics, teaching context, assessment design, feedback practices, and learner uptake interact to shape learning outcomes. This complexity is captured by Biggs’ 3P model, which conceptualises student factors, the educational environment, and learning approach as dynamically interacting components, rather than a linear or unidirectional relationship [6,7].

Methodological considerations, limitations, and future direction

This study used the Biggs R-SPQ-2F to quantify deep and surface learning approaches [7]. Its relevance is supported by a recent systematic review of 49 studies in health professions education examining relationships between learning approaches, teaching methods, and assessment outcomes. Of these, 38% used the R-SPQ-2F, matching the proportion that employed the Approaches and Study Skills Inventory for Students (ASSIST), while the remaining studies used six other psychological instruments [29]. Consistent with foundational theory [5,8], this study does not classify students as surface or deep learners, but recognises that learners may adopt both approaches to varying degrees. This was reflected methodologically by including both deep- and surface-approach scores as covariates in the linear mixed-effects model.

Students completed the R-SPQ-2F at baseline, and all assessments were conducted within three months to minimise potential shifts in learning approach. Although learning approaches may be influenced by the educational environment, Biggs’ framework also recognises student predispositions [5,7], and longitudinal evidence from medical students suggests a degree of stability over time [30]. Nevertheless, some change in learning approach during the study period cannot be excluded.

Although the R-SPQ-2F does not measure the strategic learning approach, it was selected because the study focused specifically on deep and surface learning as psychological constructs in relation to AF. The strategic approach, representing flexibility in switching between deep and surface modes under high-stakes pressure [29], was less directly relevant here, as the assessments studied were part of a broader year-long formative assessment programme and contributed only modestly to overall summative grading.

The within-student longitudinal design across eight physiology topics broadened content sampling and allowed DA and MCQA performance to be compared within the same students, thereby reducing confounding by stable between-student differences. Nevertheless, the study was conducted within a single institution and cohort, which may restrict generalisability, and its observational design precludes causal inference. The analysis examined whether baseline learning-approach scores were differentially associated with subsequent marks across AFs; it did not test whether AFs changed students’ learning approaches or whether learning approach causally determined performance. Unmeasured factors such as prior academic attainment, motivation, attendance, language proficiency, writing fluency, or general academic ability may therefore have influenced both learning approach and performance.

As the assessments were formative and low-stakes, students may not have prepared uniformly, potentially leading to an underestimation of performance. However, because each student completed both DAs and MCQAs for each topic in the same sitting, preparedness was likely to be broadly comparable across formats within each student.

Demographic covariates such as admission category, age, and gender were not included in the primary model. The student-level random intercept accounted for stable between-student differences in overall performance, but it does not replace direct adjustment for measured demographic or educational characteristics. Nevertheless, the distribution of admission category in the analytic cohort was similar to that of the overall class (Table 1), providing some reassurance against selection bias on this measured characteristic. Demographic data were not collected for non-respondents to the baseline questionnaire, so the mechanism underlying non-response could not be formally assessed.

DA and MCQA items for each topic were drawn from the institution’s summative assessment bank and matched on construct and revised Bloom’s taxonomy level through a three-round Delphi process with predefined consensus criteria. However, this process addressed the intended cognitive level of each item pair and does not guarantee that the cognitive processes enacted by students were equivalent across formats. Because items were sourced from a summative bank, findings could partly reflect the quality of items within each format rather than the properties of the AF in general.

MCQA scripts were scored using a predetermined answer key and were therefore not dependent on marker judgement. DA scripts required examiner judgement, although scoring was standardised through an analytic rubric and model answers. To evaluate this potential source of measurement error, a post hoc inter-rater reliability audit was conducted on 218 randomly selected DA scripts, representing approximately 25% of the total. This showed strong absolute agreement between markers (ICC = 0.873, 95% CI 0.838-0.901), supporting the reproducibility of DA scoring in the audited subset. However, because the audit was post hoc, limited to a subset of scripts, and assessed inter-rater rather than intra-rater reliability, some unmeasured marker variance across the full DA dataset may remain. Such variance could have affected DA-specific associations, particularly if scoring was influenced by construct-irrelevant features such as written expression, clarity, or language quality, in addition to the intended physiological content.

The relatively small number of items per test may have contributed to lower MCQA reliability, especially since MCQs are often praised for their consistency in broader educational contexts [25]. Lower reliability in shorter MCQA tests would be expected to attenuate associations between learning-approach scores and MCQA marks through classical measurement error, which could partly account for the weaker MCQA slopes in the primary model. Additionally, the absence of negative marking may have reduced discriminating power by allowing correct responses through guessing, further attenuating associations with learning-approach scores. While the primary model assumed homogeneous residual variance across AFs, residual diagnostics did not suggest systematic heteroscedasticity: residual spread was approximately constant across predicted values, with no obvious fanning pattern, reducing concern that the format-dependent associations reflect a variance artefact.

The administration order of the two AFs was fixed within each topic; order and fatigue effects are therefore fully confounded with topic and cannot be separately estimated. The topic fixed effect absorbs average between-topic differences but cannot isolate format-specific order effects.

Although the simulation-based design sensitivity analysis suggested adequate power to detect modest AF-dependent interaction effects in the primary LMM, with 72.9-81.6% power for conservatively benchmarked interactions, precision remained limited for some format-specific estimates. This was illustrated by attenuation of the positive deep-learning slope in DAs in the random-slopes sensitivity analysis. The model-based interaction plots (Figure 2) showed no indication of threshold effects or marked non-linearity across the observed range of learning-approach scores, suggesting that the linear specification adequately captured the association in this sample. Topic main effects were non-significant in the primary model, and the same interaction pattern was replicated in the Deep-minus-Surface sensitivity analysis using an alternative parameterisation of the learning approach. While neither test directly examines whether the format-by-learning-approach interaction varies across individual topics, this consistency provides indirect reassurance that the observed pattern was not driven by one or two topics.

Future studies should extend this work across multiple institutions and cohorts, include measured covariates such as prior academic attainment, attendance, and language- or writing-related variables, and prospectively strengthen assessment design through counterbalanced AF order, broader double-marking of constructed responses, and more detailed item-level psychometric evaluation. Repeated measurement of learning approach and response-process evidence, such as response times or think-aloud protocols, would also help clarify whether different AFs are associated with changes in learning approach or with different cognitive processes during assessment.

## Conclusions

In this cohort, assessment format moderated the association between learning approach and assessment performance in undergraduate physiology. In the primary LMM, DAs showed clearer differentiation by learning approach than MCQAs: higher deep-learning scores were associated with slightly higher DA marks, and higher surface-learning scores with lower DA marks, whereas MCQAs showed little evidence of association with either learning-approach dimension. DAs therefore differentiated variation in learning approach more clearly than MCQAs in this setting. Evidence for the negative association with surface learning was more robust across models than evidence for a positive association between deep learning and DA performance, which was attenuated in the random-slopes sensitivity analysis.

These findings suggest that DAs may provide more discriminating information about variation in learning approach than MCQAs in undergraduate medical students within formative programmatic assessment for physiology. They do not show that MCQAs inherently reward surface learning, nor do they imply that assessment format alone determines educational value. Because the educational consequences of assessment format depend not only on score patterns but also on how feedback is interpreted and used, meaningful feedback remains an important consideration in assessment design. Future multi-institutional studies that randomise administration order, strengthen equating of item difficulty and cognitive demands across formats, and incorporate direct process measures such as response-time data or think-aloud protocols would help clarify when and why different formats differentiate learning-relevant competencies.
